# Plant-Derived Natural Products as Sources of Anti-Quorum Sensing Compounds

**DOI:** 10.3390/s130506217

**Published:** 2013-05-13

**Authors:** Chong-Lek Koh, Choon-Kook Sam, Wai-Fong Yin, Li Ying Tan, Thiba Krishnan, Yee Meng Chong, Kok-Gan Chan

**Affiliations:** 1 Natural Sciences and Science Education AG, National Institute of Education, Nanyang Technological University, 1 Nanyang Walk, 637616, Singapore; E-Mails: chonglek.koh@nie.edu.sg (C.-L.K.); choonkook.sam@nie.edu.sg (C.-K.S.); 2 Division of Genetics and Molecular Biology, Institute of Biological Sciences, Faculty of Science, University of Malaya, Kuala Lumpur 50603, Malaysia; E-Mails: yinwaifong@yahoo.com (W.-F.Y.); etaly87@yahoo.com (L.Y.T.); thibavengdes@yahoo.com (T.K.); cymbaby_85@hotmail.com (Y.M.C.)

**Keywords:** anti-infective, autoinducer, bacterial cell-to-cell communication, biofilm, infectious diseases, *N*-acylhomoserine lactones, pathogen, quorum quenching, quorum sensing, virulence factors

## Abstract

Quorum sensing is a system of stimuli and responses in relation to bacterial cell population density that regulates gene expression, including virulence determinants. Consequently, quorum sensing has been an attractive target for the development of novel anti-infective measures that do not rely on the use of antibiotics. Anti-quorum sensing has been a promising strategy to combat bacterial infections as it is unlikely to develop multidrug resistant pathogens since it does not impose any selection pressure. A number of anti-quorum sensing approaches have been documented and plant-based natural products have been extensively studied in this context. Plant matter is one of the major sources of chemicals in use today in various industries, ranging from the pharmaceutical, cosmetic, and food biotechnology to the textile industries. Just like animals and humans, plants are constantly exposed to bacterial infections, it is therefore logical to expect that plants have developed sophisticated of chemical mechanisms to combat pathogens. In this review, we have surveyed the various types of plant-based natural products that exhibit anti-quorum sensing properties and their anti-quorum sensing mechanisms.

## 1. Introduction

**Figure 1.**
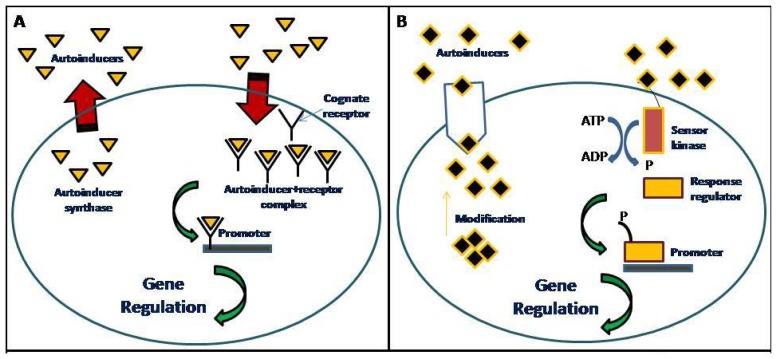
A graphic diagram of QS molecular signaling network of (A) Gram-negative bacteria: the autoinducers [ 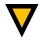] are produced and diffused freely out of the cell. When the concentration of the autoinducers reach the threshold value, a positive feedback loop will be formed that causes more autoinducers to be synthesized. The autoinducers produced will bind to their cognate receptor [ 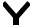] to form an autoinducer-receptor complex [ 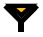] which will then binds to the target promoter that lead to QS gene regulation; and (B) Gram-positive bacteria: the precursor peptide autoinducers [ 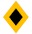] are modified and transported out of the cell by ATP-binding cassette exporter complex. When the concentration of the peptide autoinducers reach the threshold value, the sensor kinase protein will be activated and phosphorylate the response regulator protein, which will then binds to the target promoter that will lead to QS gene regulation.

Communication among bacteria is achieved via the production, diffusion, detection and responses to chemical signaling molecules known as autoinducers. When a threshold concentration is reached, the autoinducers are detected and this leads to quorum sensing (QS) gene regulation (Figure 1). This QS process uses autoinducers for the regulation of bacterial behaviors such as formation and release of virulence factors, antibiotic production and also biofilm formation [1,2]. Alternative QS concepts, such as diffusion sensing and efficiency sensing have been previously reported [3,4]. There are several QS systems used by bacteria. For example, Gram-positive bacteria primarily use the oligopeptide signaling systems whereas Gram-negative bacteria primarily uses the LuxR/I-type QS system [5].

Due to the extensive emergence of antibiotic-resistant bacteria, there is a rising need to control this drug resistance. Many opportunistic pathogenic bacteria depend on QS systems to coordinate their virulence expression. Thus, interference with QS has been regarded as the novel way to control bacterial infections [6]. QS is an evident target to capably block QS signals by inducing attenuation of pathogens' virulence determinants. It is believed that as bacterial growth is not affected, inhibition of QS does not impose selective pressure for the development of resistance [7,8]. However, this point has been challenged on the possibility of resistance to QS disruption might occur [9]. For example, a well studied QS antagonist, brominated furanone C-30, causing rapid bacterial resistance due to mutation has been reported recently [10].

## 2. Plants as QS Antagonists?

Natural products were explored initially because of their therapeutic values in traditional medical practice, but recently, there is has been an increasing interest in the biological function and therapeutic roles of natural products and their ecological role in regulating interactions between microorganisms. It is speculated that unlike human and mammals that possess immune systems to defend against invaders, plants are lacking such sophisticated immunity to ward off invading pathogens, so instead of relying on cellular and biochemical defense systems, plants may have evolved to produce anti-QS compounds that can be used to defeat QS pathogens.

To date, biologically active constituents of natural products, especially plant-derived ones, have led to the discovery of new drugs used for treatment of numerous diseases [11]. Various studies showed that eukaryotes have evolved efficiently to manipulate bacterial QS systems and protect themselves from pathogen attack [12]. An important discovery was the fact that the halogenated furanones produced by the marine red alga *Delisea pulchra* interfere the *N*-acylated homoserine lactone (AHL) regulatory system in several Gram-negative bacteria. These furanones actually enable the alga to influence the bacterial colonization and fouling on its own surface in the natural marine environment [13,14]. Earlier studies have also reported that certain bacteria possess the ability to quench QS. For example, an *N*-acylhomoserine (AHL) lactonase enzyme, AiiA from *Bacillus sp.*, was found to hydrolyze the lactone bond of the AHL signalling compound [15]. Interestingly, the paraoxonase (PON) enzymes in human airway epithelial cells also show interference with QS systems [16].

In this review, we will summarize the QS antagonists derived from plant sources and also provide better understanding of the interference of QS by these antagonists. Although this review represents an evaluation of publicly available data, there may be some important antagonists, especially the non-plant-based compounds that have been excluded.

## 3. Examples of QS Antagonists

In recent years, the discovery of QS antagonists of bacterial and non-bacterial origin has increased tremendously. There are some chemically synthesized compounds that inhibit QS, but most of the antagonists have been discovered in plants extracts. Since these plants can be consumed by humans, the active compounds that are having QS inhibitory activities from the plants should be deemed as safe and should not cause toxicity towards human cells, but toxicity studies on these compounds are still necessary. Not only to plants produce mimicry molecules that are anti-QS, but certain plant parts such as pea (*Pisum sativum*) seedlings [17] also produce exudates that contain compounds that can interfere with QS. These findings provide a better understanding of how the compounds inhibit bacterial QS systems. Biosensors such as *Escherichia coli* [pSB401], *E. coli* [pSB1075], *Chromobacterium violaceum* CV026 are used by researchers to aid the screening for compounds/extracts with anti-QS abilities. These biosensors do not possess the ability to produce any AHLs. External AHLs are supplied exogenously to induce QS traits such as bioluminescence and violacein production which can be quantified. The anti-QS ability of compounds/extracts are measured by the significance of the inhibition. Table 1 shows some of the antagonists discovered in recent studies.

## 4. Mechanisms

Natural products play a pivotal role for treating and preventing infectious diseases [44]. The plant compounds usually target the bacterial QS system via three different ways, by either stop the signaling molecules from being synthesized by the *luxI* encoded AHL synthase, degrading the signaling molecules and/or targeting the luxR signal receptor [45].

AHL biosynthesis typically involves a series of reactions that use *S*-adenosyl methionine (SAM) as the amino donor to generate the homoserine lactone ring moiety, and an acyl carrier protein (ACP) as the precursor for the *N*-acyl side chain of the AHL molecules. With this knowledge, several SAM analogues have been synthesized and show anti-QS activity [46]. However, based on our experience on extensive screening of hundreds of plant extracts to specifically look for luxI synthase activity, we did not succeed in finding any suggesting that it is rather rare for plants to possess such anti-luxI synthase activity (unpublished data).

**Figure 2.**
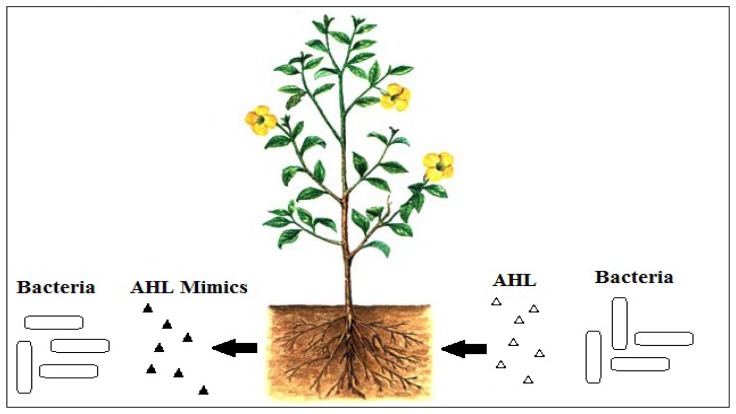
A schematic figure which shows the QS interactions between the plants and the bacteria. Bacteria release AHL molecules [△] which can cause virulence such as biofilms and crown gall formation. The plants, on the other hand, will response to the bacteria signaling molecules by releasing AHL mimic compounds [▲] for defense purposes and this will then affect the bacterial QS system.

To interfere with signal reception, there can be competitive and non-competitive molecules that can interfere with the binding of AHL to its cognate LuxR receptor. It is logical to imagine that for competitive molecules to bind to AHL receptor, these molecules must be structurally similar to AHLs. For non-competitive binding to the AHL receptor, conceivably, these molecules will bind to the site on the receptor other that than the AHL binding site. Plants can produce molecules that structurally mimic the AHLs [17], and such competitive binding is effective to block activation of AHL-mediated QS (Figure 2). In addition to that, QS inhibitors can also affect the integrity of biofilms and thus, will make the bacteria more susceptible to conventional antibiotics [47]. This serves as an advantage as it can help to minimize the possibility of the bacteria from becoming resistant [48].

One of the most extensively studied natural products is the red marine alga known as *D. pulchra*. This Australian seaweed appears to be able to control bacterial colonization by interfering with the AHL system [49]. Halogenated furanones were found to inhibit the QS regulated behaviors by competitively bind to the LuxR type proteins [50] and thus, promote their rate of proteolytic degradation without killing the bacteria [51]. Previous research has also shown that furanones are strong inhibitors for both AI-1- and AI-2-mediated QS systems [50] and such inhibition is partially relieved when the AHL concentration increases [49]. Besides, these particular compounds have been reported to reduce the ability of the *E. coli* cells to produce biofilms by inhibiting AI-2 activity [52]. Furanones also play a very important role in decreasing the light emission among the *Vibrio* species, hindering the pigment production in *C. violaceum* and stop the swarming motility in *S. liquefaciens* [49,53,54].

Besides *D. pulchra*, grapefruit extract also contains some bioactive compounds such as furocoumarins, carotenoids, limonoids, pectin and coumarin that have antibacterial and antifungal activities [55]. Furocoumarins were shown to have strong inhibition against both AI-1 and AI-2 activities, as well as hinder the formation of biofilm in *E. coli*, *S. typhimurium* and *P. aeruginosa* [56]. In addition to that, obacunone has been proven to have a strong antagonistic activity against both AHL and AI-2 systems, biofilm formation and EHEC virulence [57].

Our group has recently reported a non-competitive compound namely malabaricone C whose structure is not similar to AHL but possesses anti-QS activity. Malabaricone C is extracted from nutmeg (*Myristica cinnamomea*) where it successfully inhibits both the lasR and rhlR systems in *P.aeruginosa* PA01and also CviR in *C. violaceum* [41] and does not inhibit AHL production in *P.aeruginosa* PA01. Extracts of propolis have also been proven to inhibit the production of violacein in *C. violaceum*, as well as the LasA and LasB protease activities in *P. aeruginosa* [58].

There are also other higher plants such as vegetables that are found to possess anti-QS properties [17]. The examples include carrot, chamomile, and water lily as well as an array of peppers that have been proven to have anti-QS activity against the luxI-gfp reporter strain. Previous research has reported that metabolites such as disulphides and trisulphides which are extracted from garlic can inhibit LuxR-based QSI in *P. aeruginosa* [59]. Rosmarinic acid extracted from sweet basil can decrease the expression of the elastase and protease, as well as biofilm formation in *P. aeruginosa* [60]. Pea seedlings and root exudates are also found to inhibit pigment production, exochitinase activity and protease activity in *C. violaceum* [17]. *Medicago truncatula*, rice, tomato and soybean can also produce substances that mimic the activities of the AHL [17,61].

In addition to this, research has also proven that plants have the ability to degrade the signaling molecules produced by the bacteria and this will obstruct the bacteria virulence factors by disrupting their communication systems [15]. Plant root-associated fungi such as *Phialocephala fortinii* and *Meliniomyces variabili* and an Ascomycete isolate have been found to have the ability to degrade the AHL and have been proposed as an option for diminishing the bacterial virulence [62].

## 5. Conclusion

It is concluded that anti-QS is as important as antibacterial activity as it will unlikely cause resistance problems as it does not pose selection pressure. It is important to establish the *modus operandi* of the different QS antagonists in the pathogens in order to establish whether the antagonists are narrow or broad spectrum. Most antagonists are reported have narrow spectrum activity which may be used as a shield or sword. A narrow spectrum antagonist will only target specific pathogens where this may be useful to specifically targeting a type of pathogen in a polymicrobial environment such as those in the infection site. But on the other hand, such a narrow action antagonist may have limited clinical value. Also, the anti-QS antagonists may serve as the next generation “magic bullets”, but care must be taken that these molecules that are not bactericidal so they may have limited application for immunocompromised patients. Perhaps, a cocktail therapy involving both antibiotics and anti-QS antagonists may provide synergistic effects.

## Figures and Tables

**Table 1.** Antagonist of QS against selected bacteria and pathogens.

**Source**	**Antagonist**	**Inhibition against**	**Reference**
Plant extracts	*Melicope lunu-ankenda* (leaves) *Syzygium aromaticum* (bud)	*E. coli* [pSB401] *E. coli* [pSB1075] *C. violaceum* CV026 *P. aeruginosa* PA01 *P. aeruginosa lecA∷lux*	[18,19]
	Garlic (bulbs)	*P. aeruginosa*	[7,20]
	*Vanilla planifolia* (beans)	*C. violaceum* CV026	[21]
	*Tremella fuciformis* (whole)	*C. violaceum* CV026	[22]
	*Panax notoginseng* (flowers and roots) *Areca catechu* (seeds) *Prunus armeniaca* (kernel of seed) *Prunella vulgaris* (whole) *Nelumbo nucifera* (leaves) *Punica granatum* (bark) *Imperata cylindrical* (stem) *P. ginseng* (roots)	*C. violaceum* CV026 *P. aeruginosa* PA01	[23,24]
	*Moringa oleifera* (leaves and fruits)	*C. violaceum* ATCC 12472	[25]
	*Capparis spinosa* (fruits)	*C. violaceum* CV026 *P. aeruginosa* *E. coli* *Proteus mirabilis* *Serratia marcescens*	[26]
	*Laurus nobilis* (fruits, flowers, leaves, bark)	*C. violaceum* ATCC 12427	[27]
	*Acacia nilotica* (green pod)	*C. violaceum* ATCC 12472	[28]
	*Quercus virginiana* (leaves) *Chamaesyce hypericifolia* (aerial) *Tetrazygia bicolor* (leaves) *Conocarpus erectus* (leaves) *Bucida burceras* (leaves) *Callistemon viminalis* (leaves, inflorescence)	*C. violaceum* ATCC 12472 *C. violaceum* CV026 *Agrobacterium tumefaciens* NTL4	[29]
	*Vaccinium macrocarpon* *V. angustifolium* *Rubus idaeus* *R. eubatus* *Fragaria* sp. *Vitis* sp. *Origanum vulgare* *Rosemarinus officinalis* *Ocimum basilicum* *Thymus* sp. *Brassica oleracea* *Curcuma longa* *Zingiber officinale*	*C. violaceum* CV026 *C. violaceum* 31532 *P. aeruginosa* PA01 *E. coli* O157:H7	[30]
	*Lonicera alpigena* *Castanea sativa* *Juglans regia* *Ballota nigra* *R.officinalis* *Leopoldia comosa* *Malva sylvestris* *Cyclamen hederifolium* *Rosa canina* *R. ulmifolius*	*Staphylococcus aureus*	[31]
	*Ananas comosus* *Musa paradiciaca* *Manilkara zapota* *Ocimum sanctum*	*C. violaceum* ATCC 12472 *C. violaceum* CV026 *P. aeruginosa* PA01	[32]
	*Scutellaria baicalensis*	*C. violaceum* CV026	[33]
	*Scorzonera sandrasica*	*C. violaceum* ATCC 12472 *C. violaceum* CV026	[34]
	Orange	*Yersinia enterocolitica*	[35]
Essential oils	Tea tree Rosemary	*C. violaceum* CV026	[36]
	*Lippia alba* *Ocotea* sp. *Elettaria cardamomum* *Swinglea glutinosa* *Myntotachys mollis* *Zingiber officinale*	*P. putida* [pRK-C12) *E. coli* [pJBA132]	[37]
	*Piper bredemeyeri* (leaves) *P. brachypodom* (leaves) *P. bogotence* (whole)	*C. violaceum* CV026	[38]
Bioactive metabolites	*Phellinus igniarius*	*C. violaceum* CV026	[39]
Plant exudates	Exudates from pea (*Pisum sativum*)	*Serratia liquefaciens* MG44 *S. faciens*MG44	[17]
Fungal extracts	*Ganoderma lucidum*	*C. violaceum* CV026	[40]
Broccoli	Sulforaphane Erucin	*P. aeruginosa*	[41]
*Myristica cinnamomea* *Combretum albiflorum*	Malabaricone C Catechin	*P. aeruginosa* PA01 *C. violaceum* CV026	[42,43]
